# Treatment of endometriosis with dienogest in combination with traditional Chinese medicine: A systematic review and meta-analysis

**DOI:** 10.3389/fsurg.2022.992490

**Published:** 2022-11-01

**Authors:** Yu’e Wu, Yujie Liu, Huanhuan Jia, Chao Luo, Huan Chen

**Affiliations:** ^1^ Guangdong Laboratory Animals Monitoring Insitute, Guangdong Provincial Key Laboratory of Laboratory Animals, Guangzhou, China; ^2^ Department of Obstetrics, Zhuzhou Central Hospital, Zhuzhou, China; ^3^ Department of Neurology, Zhuzhou Central Hospital, Zhuzhou, China

**Keywords:** endometriosis, dienogest, traditional Chinese medicine, meta-analysis, review

## Abstract

**Background:**

Endometriosis is now considered to be a systemic disease rather than a disease that primarily affects the pelvis. Dienogest (DNG) has unique advantages in the treatment of endometriosis, but it also has side effects. Alternatively, Traditional Chinese Medicine (TCM) has been used for over 2000 years in the treatment and prevention of disease and growing numbers of Chinese scholars are experimenting with the combined use of Dienogest and TCM for endometriosis treatment.

**Objectives:**

This review evaluated the efficacy and safety of TCM in combination with Dienogest in the treatment of endometriosis through meta-analysis.

**Methods:**

MEDLINE, Embase, the Cochrane Library, PubMed, Web of Science, China National Knowledge Infrastructure, Journal Integration Platform, and Wanfang were used in literature searches, with a deadline of May 31, 2022. Literature quality was assessed using the Cochrane Collaboration “risk of bias” (ROB2) tool, and the “meta” package of R software v.4.1 was used for meta-analysis. Dichotomous variables and continuous variables were assessed using the relative risk (RR) and 95% confidence intervals (95% CI); standard mean differences (MD) and 95% CI, respectively.

**Results:**

Twelve human randomized controlled trials (RCTs) and one retrospective study, all 13 written in the Chinese language, were included in the meta-analysis (720 experiments and 719 controls). The result indicated that TCM plus Dienogest was superior to Dienogest/TCM alone in increasing the cure rates (RR = 1.3780; 95% CI, 1.1058, 1.7172; *P *= 0.0043), remarkable effect rate (RR = 1.3389; 95% CI, 1.1829, 1.5154; *P *< 0.0001), invalid rate (RR = 0.2299; 95% CI, 0.1591, 0.3322; *P* < 0.0001), and rate of adverse effects (RR = 0.6177; 95% CI, 0.4288, 0.8899; *P* = 0.0097). The same conclusion was drawn from the subgroup analysis.

**Conclusion:**

Results suggest that TCM combined with Dienogest is superior to Dienogest or TCM alone and can be used as a complementary treatment for endometriosis. TCMs have potential to improve clinical efficacy and reduce the side effects of Dienogest. This study was financially supported by Annual Science and Technology Steering Plan Project of Zhuzhou. PROSPERO has registered our meta-analysis as CRD42022339518 (https://www.crd.york.ac.uk/prospero/record_email.php).

## Introduction

Historically, endometriosis was thought to involve the growth of endometrial tissue outside of the uterus, typically on the lining of the pelvic cavity (peritoneum) or on the ovaries (1). However, this description is outdated and no longer reflects the true extent and manifestation of the disease. It is now considered to be a systemic disease rather than a disease that primarily affects the pelvis. For example, it can affect metabolism of the liver and adipose tissue, causing systemic inflammation and altering gene expression in the brain, leading to pain sensitivity and mood disorders (2). Endometriosis can be divided into different types, including ovarian endometriosis, peritoneal endometriosis, and deep endometriosis (3). Endometriomas are the most common form of the disease, occurring in 25%–35% of patients with endometriosis (4). It commonly occurs in association with retrograde menstruation, which usually results in endometrial tissue growing outside the uterine body (5). Treatment for endometriosis includes surgery, medical therapy, and assisted reproductive technology (ART) (6). While surgical resection of endometriotic lesions is considered the standard therapeutic approach in symptomatic endometriosis, recurrence of the disease and symptoms following surgery is common and often requires repeated surgery (7). The pharmacological treatment of endometriosis to achieve maintenance includes GnRH agonists, steroid contraceptives, progestins (orally and intrauterine), and aromatase inhibitors (8). As research progresses, the current view is that medication should be the primary treatment option for patients with pelvic pain who have no immediate desire to conceive; for specific infertility patients, assisted reproductive technology can be performed without surgery (6). In spite of the numerous health implications of endometriosis and extensive research efforts, current medical treatments such as GnRH analogs, oral contraceptives, and progestins are often ineffective or cause significant side effects (9). In the absence of long-term treatment with safe medication, recurrence can necessitate hysterectomy and bilateral oophorectomy (10).

Dienogest (DNG), a 19-nortestosterone derivative, has good bioavailability and a strong progestational effect because of its high selectivity by progesterone receptors (11). A growing body of research has demonstrated the unique advantages of DNG in the treatment of endometriosis: Patients treated with DNG after conservative surgery for endometriosis had a significantly lower risk of postoperative disease recurrence than those with expecting treatment (12); long-term use of DNG for rectosigmoid endometriosis can relieve symptoms (13); a significant increase in improvement in endometriosis lesions, pain symptoms, and quality of life were found in women taking DNG compared to women on continuous combined oral contraceptives (14); it is also an effective drug for controlling the pain symptoms associated with deep infiltrating endometriosis (DIE), even without reducing the volume of DIE nodules (15); it can be used for a long time and is well tolerated (16). DNG has therefore been recommended as an alternative method of controlling symptoms associated with endometriosis (17) and as maintenance therapy for patients with endometriosis to reduce the rate of disease recurrence after conservative surgery (18). However, the most common side effects of using DNG include irregular vaginal bleeding, weight gain, and headaches (19).

Chinese medicine is a collective term for traditional Chinese medicines derived from plants, animals, minerals, and their finished products, as well as modern medicines produced with modern technology under the guidance of Chinese medical theory (20). These include traditional Chinese medicine (TCM) or TCM formulas, and have been used to treat and prevent diseases for over 2000 years (21). In China, TCM has gained growing popularity and includes not just single herbs, but Chinese medicinal compounds, Chinese patent medicines (CPMs), and acupuncture (22). The most popular application of TCM is Chinese herbal medicine (CHM), which consists of sliced herbs and Chinese patented drugs (23). It is usually prepared as a formula by unique methods using a combination of herbs (24). CPMs are widely used as a substitute and adjuvant to western medicines (25). They are one of the most important parts of TCM used in clinical practice as derivatives of Chinese herbal medicine (26). Modern pharmacological research has led to the use of greater numbers of Chinese medicine extracts in modern pharmaceutical preparations, including oral CPM (OCPM) and Chinese medicine injection (27). In traditional CPM (TCPM), Chinese medicines are used as raw materials; the product is then created based on the prescription and preparation technology to prevent and treat disease (28). TCM is an alternative treatment for endometriosis in China owing to its significant therapeutic effect and low toxicity (29, 30). Greater numbers of Chinese scholars are now experimenting with the combined use of DNG and TCM for the treatment of endometriosis.

Therefore, this review set out to evaluate the efficacy and safety of TCM in combination with DNG in the treatment of endometriosis through a meta-analysis to provide evidence for its use in clinical practice.

## Materials and methods

### Data sources and search strategy

MEDLINE, Embase, the Cochrane Library, PubMed, Web of Science, China National Knowledge Infrastructure (CNKI), Journal Integration Platform (VIP), and Wanfang were utilized to perform the literature search. Publication was limited to English and Chinese. The time deadline was May 31, 2022. The retrieval strategy was based on subject words and free words and the search terms: (Endometriosis OR Endometrioses OR Endometrioma OR Endometriomas) AND (Chinese Traditional Medicine OR Chinese herbal drugs OR Chinese Plant Extracts or Zhong Yi Xue) AND (Dienogest OR STS-557) were used as keywords.

### Inclusion and exclusion criteria

The following inclusion criteria were used: the intervention group was treated with a combination of TCM and DNG, and the control group was treated with TCM or DNG alone, in studies with similar hypotheses and study methods; studies with years of conduct or publication; studies with clearly defined sample sizes; studies with clear criteria for patient selection and case diagnosis, and clear measures of intervention and control; count information available as OR/RR or calculated from the number of cases in the sample and the number of incidences, and measure information available as mean, standard deviation, and sample size. Exclusion criteria included: duplicate reporting; flawed study design and poor quality; incomplete data and unclear outcome effects; incorrect statistical methods that could not be corrected; OR/RR, number of sample cases and number of incidence cases not available for count data or when the mean, standard deviation, and sample size were not available for measure data. Case reports, pedigree studies, case series, editorials, reviews, expert opinions, animal studies, and meta-analyses were also excluded in the present meta-analysis. Research abstracts were independently reviewed by two researchers, and eligible literature was intensively read for further study.

### Data extraction

According to the inclusion and exclusion criteria, two investigators (YW and YL) independently selected the literature. Extractions included general information such as first author, publication year, and number of participants in the treatment group and control group, age, course of disease, and outcomes of patients. There were two types of outcome indicators, one for dichotomous variables and one for continuous variables. The former included the rates of treatment effect, the rate of adverse effects; the latter included estradiol (E2), progesterone (P), luteinizing hormone (LH), follicle stimulating hormone (FSH), cancer antigen 125(CA-125), cancer antigen 199(CA-199), matrix metalloproteinase 2(MMP-2), matrix metalloproteinase 9(MMP-9), Galectins-3(Gal-3), vascular endothelial growth factor (VEGF), cyst size, visual Analogue Scale/Score (VAS), ovulation recovery time, menses recovery time, and recurrence rate. However, the main outcome indicators assessed were the rates of treatment effect and the rate of adverse effects. Any disagreements were resolved through discussion and, if necessary, by engaging a third senior author (HJ).

### Quality assessment

For the assessment of literature quality, using the Cochrane Collaboration “risk of bias” (ROB2) tool, we assessed ROB. Two researchers independently (YW and YL) evaluated the quality of the literature. Five bias domains were included: randomization process, deviations from intended interventions, missing outcome data, measurement of the outcome, selection of the reported result, and overall bias.

### Statistical analysis

R software v.4.1 was used for data analysis, and the “meta” package (31) for meta-analysis. To evaluate dichotomous variables, we used the relative risk (RR) and 95% confidence intervals (95% CI). For continuous variables, we used the standard mean differences (MD) and 95% CI. Using *I*^2^ statistics, we checked the degree of statistical heterogeneity among the included studies in accordance with the guide judgment *I*^2 ^< 50% indicating low heterogeneity whereas *I*^2^ > 50% reflected obvious heterogeneity. If the *I*^2^ statistic was >50%, then a random-effects model was used. Otherwise, a fixed-effects model was used. Analyses of subgroups were performed using the Q-test for heterogeneity. The leave-one-out method was used to determine sensitivity.

## Results

### Literature inclusion

**Figure 1:**
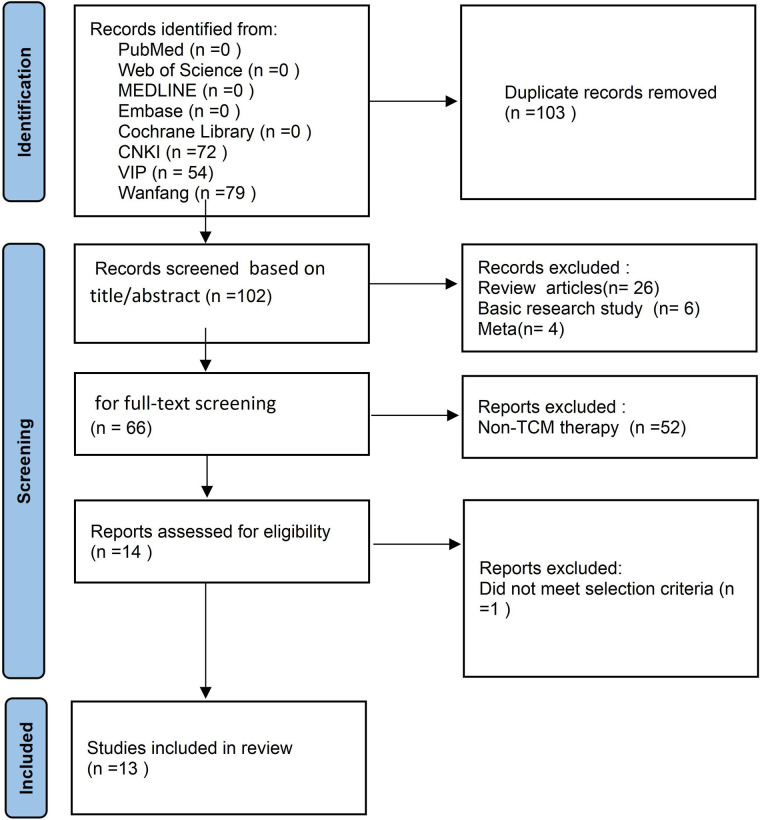
The workflow of this study.

The flow diagram for PRISMA search and selection of literature is shown in Figure 1. A total of 205 articles were identified in the initial search. Of these, 102 articles remained after removing duplicates. We excluded 36 irrelevant articles based on title and abstract screening. Following a review of the full text of the publications, 52 publications were excluded because they were not treated with TCM, another one publication was excluded because it was a comparative study between DNG and TCM.

As a result, 12 RCTs and one retrospective study, all in the Chinese language, were included in the meta-analysis (720 experiments and 719 controls). Of these studies, four involved Gui Zhi Fu Ling capsules/wan combined with DNG, three involved Gui'e lengwu decoction combined with DNG, three involved CHM combined with DNG (one of the CHMs has a composition similar to that of Gui'e lengwu decoction), and each of San Jie Zhen Tong capsules, Kuntai capsule, Jingtong yushu granule combined with DNG treatment, respectively. Table 1 displays the characteristics of the included studies. Table 2 shows the levels of outcome indicators in the two groups before treatment. In general, there were no statistically significant baseline differences between the intervention and control groups (*P* > 0.05).

**Table 1:** Characteristics of included studies.

Study	Research Type	Sample size (EG/CG)	Age^a^		Course of Disease^a^		Experiment	Control	Duration (months)	Outcomes
			EG	CG	EG	CG				
ZJ (2021) (32)	RCT	43/43	34.28 ± 4.76	34.15 ± 4.72	4.31 ± 0.57	4.4 ± 0.59	DNG + JTYS	DNG	6	EP,VAS
WQF (2021) (33)	RCT	30/30	38.55 ± 3.43	38.62 ± 3.35	8.43 ± 2.58	8.23 ± 2.12	DNG + GZFL	GZFL	3	EP,E2,P,LH,FSH,CA125,CA199,MPP2,Gal-3,VEGF
DLL (2021) (34)	RCT	47/47	38.62 ± 3.37	38.57 ± 3.42	8.57 ± 2.36	8.65 ± 2.43	DNG + SJZT	SJZT	6	EP,E2,P,LH,FSH,CA125,MPP2,Gal-3,VEGF,MPP9
ZYH (2015)^b^ (35)	RCT	63/63	23.1 ± 4.9	22.0 ± 4.1			DNG + TCM	DNG	6	EP,ORT,MRT,RR
MD (2017) (36)	RCT	40/40	33.97 ± 6.43	33.51 ± 6.82	22.58 ± 19.84	22.12 ± 19.45	DNG + GELW	DNG	3	EP,CA125,CS
WM (2019) (37)	RCT	85/85	35.3 ± 2.6	36.8 ± 2.5	5.3 ± 1.7	6.2 ± 1.5	DNG + GELW	DNG	6	EP,CA125,CS,VAS
TYY (2021) (38)	RCT	50/50	28.5 ± 5.2	28.4 ± 5.4			DNG + GZFL	DNG	3	EP,E2,P,LH,FSH,MPP2,Gal-3,MPP9
BXH (2020) (39)	RCT	53/53	31.7 ± 2.6	31.2 ± 2.8	4.7 ± 0.6	4.4 ± 0.8	DNG + GELW	DNG	6	EP,CA125,CS,VAS
LN (2020) (40)	RCT	88/87	29.04 ± 6.31	28.93 ± 6.37	11.94 ± 3.13	12.05 ± 2.98	DNG + GZFL	DNG	3	EP,E2,P,LH,FSH,CA125,CA199,MPP2,Gal-3,VEGF,MPP9,CS,VAS
JX (2021) (41)	RCT	88/89	28.87 ± 4.67	29.01 ± 4.16	5.87 ± 1.02	5.02 ± 1.44	DNG + TCM	DNG	6	EP,ORT,MRT,RR
ZWX (2021) (42)	RCT	38/37	31.87 ± 5.49	31.95 ± 5.30	3.14 ± 0.52	3.21 ± 0.48	DNG + TCM	DNG	3	EP,CA125,CA199,CS,VAS
ZYY (2021) (43)	RCT	30/30	36.5 ± 1.1	36.1 ± 0.9	2.2 ± 0.3	2.1 ± 0.1	DNG + GZFL	DNG	3	E2,P,LH,FSH,CS,VAS
LDP (2022) (44)	RET	65/65	29.62 ± 5.38	29.53 ± 6.47	1.25 ± 0.25	1.25 ± 0.75	DNG + KT	DNG	1	E2,LH,FSH,CA125

RCT, randomized controlled trial; RET, retrospective study; DNG, Dienogest; EG, experiment group; CG, control group; EP, effective percentage; E2, estradiol; P, progesterone; LH, luteinizing hormone; FSH, follicle-stimulatinghormone; CA125, cancer antigen 125; CA199, cancer antigen 199; MPP2, matrix metalloproteinase 2; MPP9, matrix metalloproteinase 9; Cal-3, Galectins-3; VEGF, Vascular endothelial growth factor; CS, Cystic size; VAS, visual Analogue Scale/Score; ORT, oviating recovery time; MRT, menses recovery time; RR, recurernce rate; JTYS, Jingtongyushu granule; GZFL, Guizhifuling capsule/wan; SJZT, Sanjiezhentong capsule; TCM, Chinese traditional medicine; GELW, Guielengwu decoction; KT, Kuntai capsule.

^a^ There was no statistical difference between the two groups.

^b^ Surgical treatment.

**Table 2:** Comparison of indicators before treatment.

Index	Author (year)	Experiment		Control		Sample size (EG/CG)			
		X	S	X	S		Model	*I* ^2^	*P*
E2 (pmol/L)	WQF (2021)	169.88	17.56	170.02	16.98	30/30	Fixed	0.0%	0.7103
	DLL (2021)	170.69	17.71	170.81	17.68	47/47			
	TYY (2021)	172.12	16.78	172.57	16.34	50/50			
	LN (2020)	170.79	17.79	171.76	17.85	88/87			
	ZYY (2021)	170.78	17.74	171.74	17.83	30/30			
	LDP (2022)	344.25	70.46	344.42	70.33	65/65			
*P* (nmol/L)	WQF (2021)	0.84	0.19	0.85	0.2	30/30	Fixed	0.0%	0.5479
	DLL (2021)	0.85	0.24	0.86	0.18	47/47			
	TYY (2021)	0.94	0.23	0.93	0.24	50/50			
	LN (2020)	0.87	0.22	0.84	0.25	88/87			
	ZYY (2021)	0.85	0.19	0.82	0.21	30/30			
LH (IU/L)	WQF (2021)	6.42	1.69	6.39	1.72	30/30	Fixed	0.0%	0.9209
	DLL (2021)	6.41	1.72	6.42	1.69	47/47			
	TYY (2021)	7.56	1.89	7.47	1.91	50/50			
	LN (2020)	6.29	1.79	6.39	1.95	88/87			
	ZYY (2021)	6.27	1.78	6.37	1.93	30/30			
	LDP (2022)	8.25	2.74	8.33	2.62	65/65			
FSH (IU/L)	WQF (2021)	6.74	2.22	6.73	2.19	30/30	Fixed	0.0%	0.8343
	DLL (2021)	6.73	2.19	6.71	2.17	47/47			
	TYY (2021)	6.75	2.01	6.81	1.98	50/50			
	LN (2020)	6.51	2.29	6.74	2.23	88/87			
	ZYY (2021)	6.49	2.27	6.72	2.21	30/30			
	LDP (2022)	12.15	2.82	12.33	2.77	65/65			
CA125 (IU/ml)	WQF (2021)	72.39	13.68	73.67	13.55	30/30	Fixed	0.0%	0.4584
	DLL (2021)	72.41	13.75	73.53	13.24	47/47			
	MD (2017)	71.56	22.95	72.24	19.32	40/40			
	WM (2019)	72.36	13.84	75.49	10.54	85/85			
	BXH (2020)	73.68	13.44	74.14	13.25	53/53			
	LN (2020)	60.29	7.54	59.85	6.78	88/87			
	ZWX (2021)	73.42	11.65	74.85	12.03	38/37			
	LDP (2022)	46.65	15.25	46.32	15.44	65/65			
CA199 (IU/ml)	WQF (2021)	61.81	7.45	61.76	7.52	30/30	Fixed	0.0%	0.8220
	LN (2020)	61.79	7.41	62.15	7.39	88/87			
	ZWX (2021)	75.08	12.16	74.86	11.98	38/37			
MPP2 (µg/L)	WQF (2021)	225.74	42.53	226.12	43.75	30/30	Fixed	0.0%	0.9232
	DLL (2021)	226.85	42.67	227.12	46.43	47/47			
	TYY (2021)	228.13	47.12	227.99	47.45	50/50			
	LN (2020)	228.54	48.35	227.13	48.76	88/87			
Gal-3 (ng/L)	WQF (2021)	7.91	3.65	7.92	3.57	30/30	Fixed	0.0%	0.9691
	DLL (2021)	7.85	3.57	7.82	3.63	47/47			
	TYY (2021)	7.93	2.42	8.01	2.24	50/50			
	LN (2020)	7.95	3.72	7.81	3.64	88/87			
VEGF (pg/ml)	WQF (2021)	166.58	112.43	165.64	110.87	30/30	Fixed	0.0%	0.9744
	DLL (2021)	166.75	113.24	166.57	111.98	47/47			
	LN (2020)	166.63	114.95	167.84	114.28	88/87			
MPP9 (ng/L)	DLL (2021)	941.53	343.64	942.47	321.85	47/47	Fixed	0.0%	0.9890
	TYY (2021)	936.12	340.23	937.01	339.57	50/50			
	LN (2020)	939.53	339.21	937.45	341.73	88/87			
Diameter (cm)	MD (2017)	3.62	0.84	3.59	0.98	40/40	Fixed	0.0%	0.0851
	WM (2019)	3.98	0.74	3.71	0.83	85/85			
	BXH (2020)	3.94	0.82	3.88	0.84	53/53			
	LN (2020)	3.13	0.47	3.07	0.48	88/87			
	ZWX (2021)	3.41	0.56	3.43	0.52	38/37			
	ZYY (2021)	3.12	0.45	3.05	0.46	30/30			
VAS	ZJ (2021)	5.46	0.54	5.51	0.56	43/43	Fixed	0.0%	0.6645
	WM (2019)	8.36	1.51	8.12	1.43	85/85			
	BXH (2020)	8.14	1.46	8.17	1.37	53/53			
	LN (2020)	6.03	1.16	5.95	1.08	88/87			
	ZWX (2021)	7.06	1.45	6.95	1.52	38/37			
	ZYY (2021)	6.01	1.14	5.93	1.06	30/30			

VAS, visual analogue scale.

### Quality assessment

**Figure 2:**
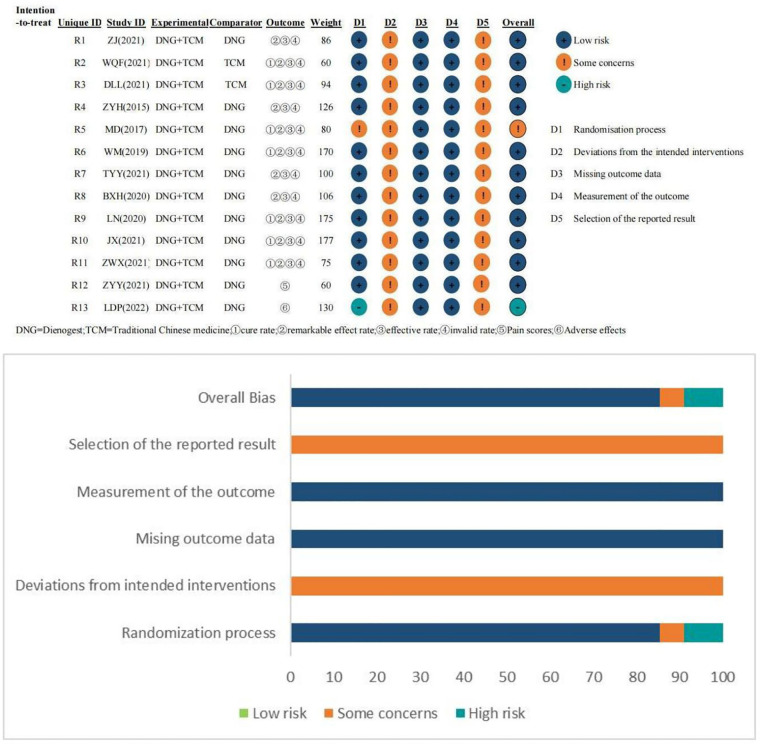
Quality assessment chart of all literature included in this study.

Figure 2 shows the results of the analysis of methodology quality of the included studies. No information regarding bias caused by deviations from the intended intervention on whether a departure occurred from the intended intervention was reported. Although the blinding of all studies to prognostic assessment was unclear, patients with endometriosis had objective indicators on rates of treatment effect or the rate of adverse effects, and it was difficult to influence prognostic assessment. In the final data analysis, all outcome data were included for randomized patients. All studies lacked clarity regarding the outcomes of blinding.

### Rate of treatment effect

**Figure 3:**
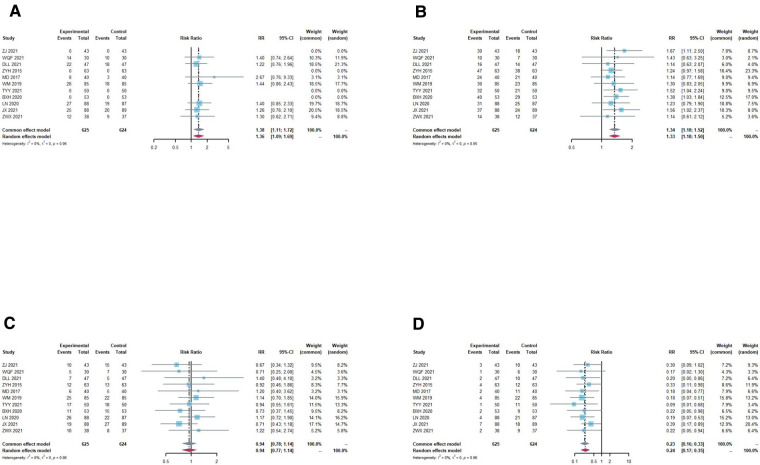
Overall meta-analysis forest plot of treatment effect rates. (**A**) cure rate, a fixed effects model was used as no significant heterogeneity (*I*^2^ = 0.0%, *P* = 0.9611) was found. The result (RR = 1.3863; 95% CI, 1.1008; 1.7458, *P* = 0.0043) indicated that TCM plus DNG was superior to DNG/TCM alone in increasing the cure rate. (**B**) remarkable effect rates, a fixed effects model was used as no significant heterogeneity (*I*^2^ = 0.0%, *P* = 0.9530) was found. The result (RR = 1.3389; 95% CI, 1.1829,1.5154; *P* < 0.0001) indicated that TCM plus DNG was superior to DNG/TCM alone in increasing remarkable effect rate. (**C**) effective rate, a fixed effects model was used as no significant heterogeneity (*I*^2^ = 0.0%, *P* = 0.8638) was found. The result (RR = 0.9411; 95% CI, 0.7752, 1.1425; *P* = 0.5395) indicated that there was no significant difference between the two groups in terms of effective rate. (**D**) invalid rate, a fixed effects model was used as no significant heterogeneity (*I*^2^ = 0.0%, *P* = 0.9629) was found. The result (RR = 0.2299; 95% CI, 0.1591, 0.3322; *P* < 0.0001) indicated that TCM plus DNG was superior to DNG/TCM alone in decreasing invalid rate.

Treatment results were classified as cure, remarkable effect, effective, and invalid. The criteria for “cure” was a decrease in the Chinese Medicine Syndromes score >90.00% and disappearance of pelvic mass; “remarkable” was a decrease in the Chinese Medicine Syndromes score of 66.67%–90.00%, a significant reduction in clinical symptoms and signs, and a reduction in pelvic mass diameter by 1/2; “effective” was a decrease in the Chinese Medicine Syndromes score of 33.33%∼66.66%, and a reduction in pelvic mass diameter by 1/3 but <1/2; “ineffective” was a decrease in the Chinese Medicine Syndromes score of <33.33%, and a reduction in pelvic mass diameter by <1/3 or aggravation. In terms of cure rate, a fixed effects model was used as no significant heterogeneity was found (*I*^2^ = 0.0%, *P *= 0.9611). The result (RR = 1.3780; 95% CI, 1.1058, 1.7172; *P *= 0.0043) indicated that TCM plus DNG was superior to DNG/TCM alone in increasing the cure rates (Figure 3A). At the same time, the intervention group outperformed the control group in terms of remarkable effect rate (Figure 3B) and invalid rate (Figure 3C), but with no significant difference between the two groups in terms of effective rate (Figure 3D).

### Rate of adverse effects

**Figure 4:**
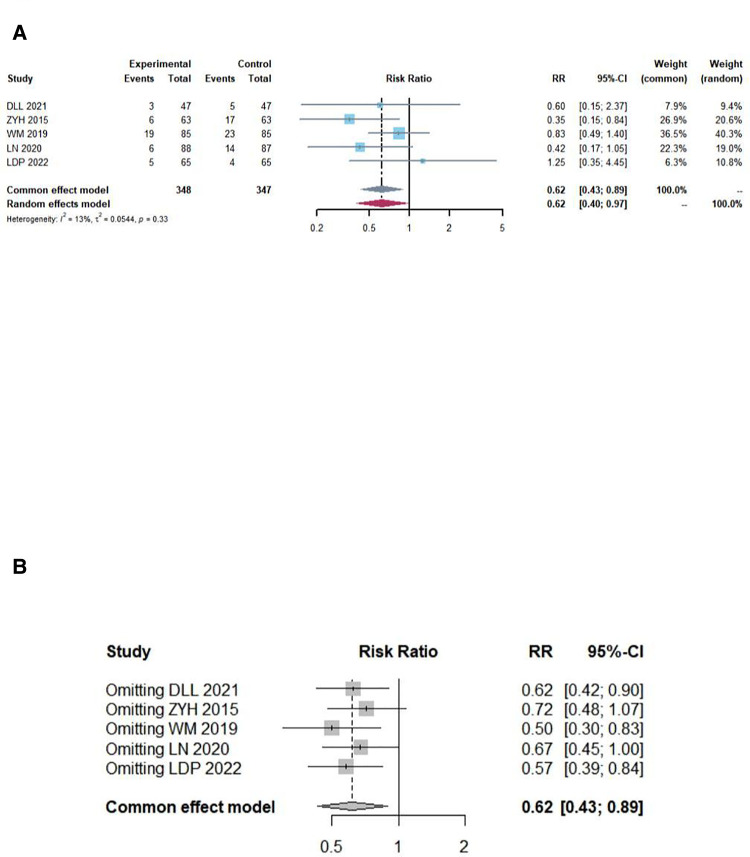
Overall meta-analysis forest and sensitivity analysis plot of adverse effects rates. (**A**) Overall meta-analysis forest plot, there were no significant statistically significant differences (*I*^2^ = 12.4%, *P* = 0.3316); thus, the fixed model was assumed. The combination of DNG with TCM for endometriosis can significantly reduce the rate of adverse reactions compared to DNG or TCM alone (RR = 0.6177; 95% CI, 0.4288, 0.8899; *P* = 0.0097). (**B**) sensitivity analysis plot of the adverse effects rates, according to the leave-one-out method sensitivity analysis, the individual study results had no impact on the meta-analysis results.

In total, five studies provided all cases of adverse effects in the experimental and control groups. In the pooled data, there were no statistically significant differences (*P* = 0.3316, *I*^2^ = 12.9%); thus, the fixed model was assumed. The combination of DNG with TCM for endometriosis significantly reduced the rate of adverse effects compared to DNG or TCM alone (Figure 4A).

### Subgroup analysis and sensitivity analysis

**Figure 5:**
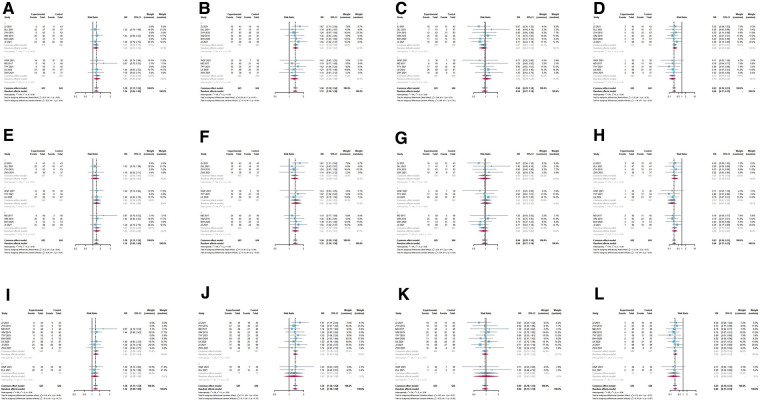
Subgroup analysis forest plot based on the treatment course, different TCM + DNG and different controls of treatment effect rates. (**A–D**) Subgroup analysis forest plot based on the treatment course of treatment effect rates. (**E–H**) Subgroup analysis forest plot based on different TCM + DNG of treatment effect rates. (**I–K**) Subgroup analysis forest plot based on different control of treatment effect rates. (**A,E,I**) cure rate; (**B,F,J**) remarkable effects rate; (**C,G,K**) effective rate; (**D,H,L**) invalid rates. Significant differences were not observed between the subgroups.

**Figure 6:**
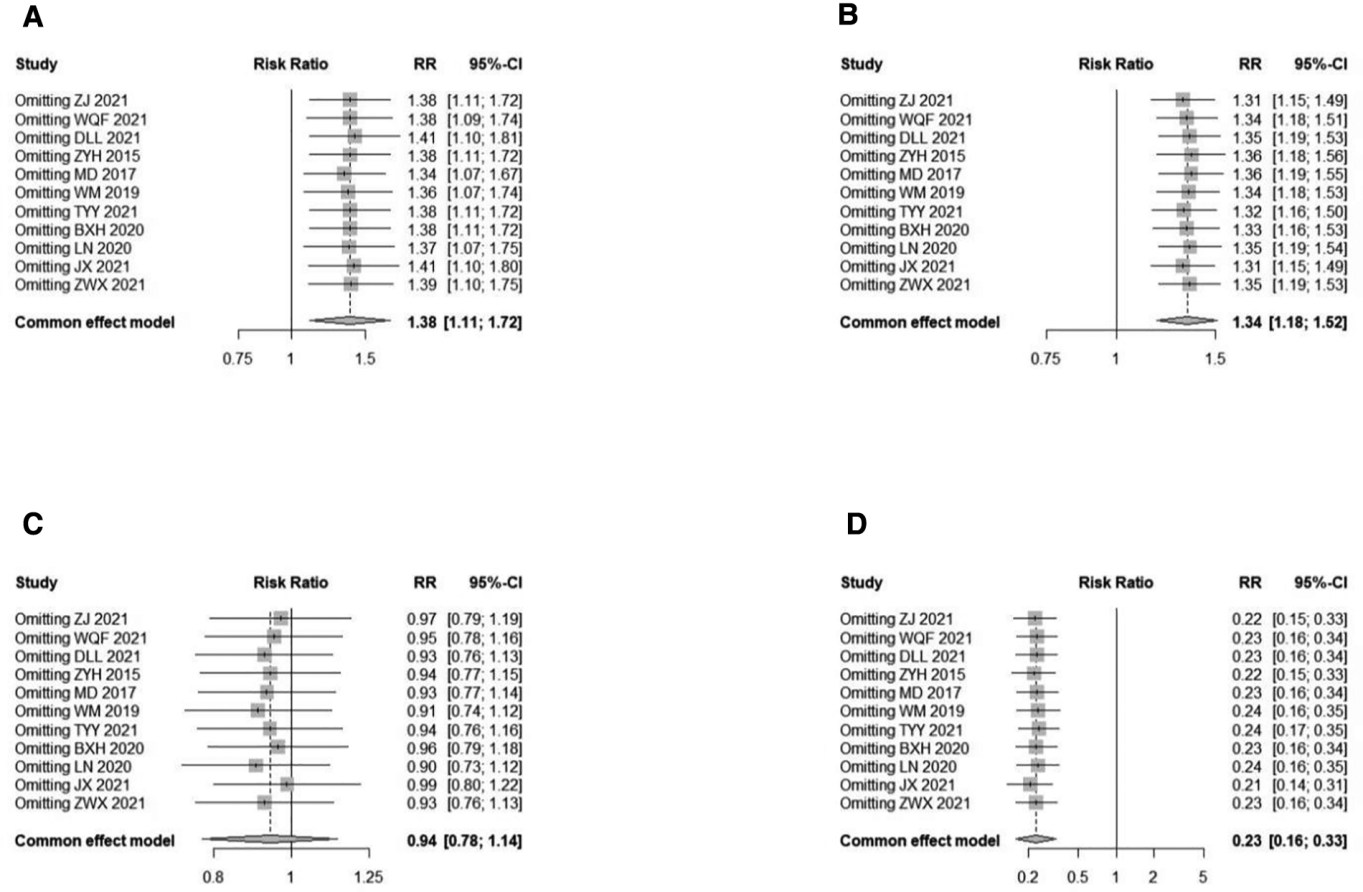
Sensitivity analysis plot of treatment effect rates. According to the leave-one-out method sensitivity analysis, the individual study results had no impact on the meta-analysis results in all four rates. (**A**) cure rate; (**B**) remarkable effect rate; (**C**) effective rate; (**D**) invalid rate.

Subgroup analysis was conducted on the cure rate, remarkable effect rate, invalid rate and adverse effects rate according to treatment duration, different TCM combined with DNG, and different control groups respectively. The combination was more effective than either drug alone (*P *< 0.05) (Figure 5). No difference was found in the subgroup analysis of the combination over different durations of treatment (Figures 5A–D). Similar results were found in the subgroup analysis of different TCM combinations with DNG (Figures 5E–H). Also, subgroup analyses with different controls had not significantly different (Figures 5I–L). According to the leave-one-out method sensitivity analysis, individual study results had no effect on the meta-analysis results of the rates of treatment effect (Figure 6) or the rate of adverse effects (Figure 4B). In most outcome evaluation indicators, individual study results had no effect on the meta-analysis results (Supplementary files).

### Publication bias

**Figure 7:**
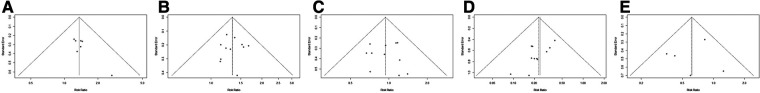
Funnel plot of treatment effect rates and adverse effects rates. (**A**) cure rate; (**B**) remarkable effect rate; (**C**) effective rate; (**D**) invalid rate; and (**E**) adverse effects rate. According to the funnel plot, there is no evidence for publication bias.

The possibility of publication bias was examined using different approaches. Figure 7 shows five funnel plots that appear visually symmetrical. In terms of cure rate, an analysis of publication bias was conducted using the trim and fill method because there were fewer than 10 included studies. The test results showed *P = *0.0058, indicating statistical significance; therefore there was publication bias in the cure rate data.

### Other outcomes

**Figure 8:**
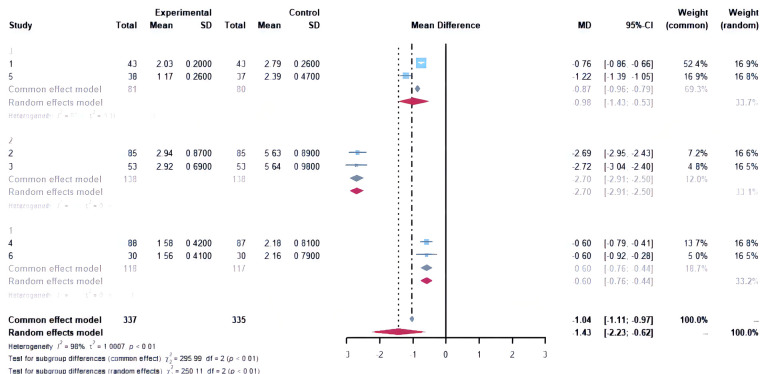
Subgroup analysis forest plot based on post-treatment visual analogue scale/score. Study 1 and 5: Jingtong yushu granule and Bushen Huayu decoction. Study 2 and 3 Gui’e lengwu decoction; study 4 and 6: Gui Zhi Fu Ling capsules/wan.

In this study, we also compared other outcome indicators after treatment and showed that the combination was significantly better than the drugs alone, in terms of E2, *P*, CA-125, CA-199, MMP-2, MMP-9, Gal-3, VEGF, cyst size, and VAS. In terms of the post-treatment VAS, subgroup analysis suggested heterogeneity stemming from Jingtong yushu granules and the Bushen Huayu decoction (Figure 8). However, we found no difference between the two groups after treatment in LH, FSH, ovulation recovery time, and menses recovery time (Table 3). The recurrence rate could not be analyzed as there was too little literature available to create a data set.

**Table 3:** Comparison of indicators after treatment.

Index	Author (year)	Experiment		Control		Sample size (EG/CG)			
		X	S	X	S		Model	*I* ^2^	*P*
E2 (pmol/L)	WQF (2021)	103.41	11.22	125.46	14.24	30/30	Random	77%	<0.0001
	DLL (2021)	103.23	11.04	125.52	14.33	47/47			
	TYY (2021)	102.12	10.12	128.12	14.21	50/50			
	LN (2020)	103.54	10.98	125.48	14.27	88/87			
	ZYY (2021)	103.52	10.94	125.46	14.25	30/30			
	LDP (2022)	125.44	30.42	172.44	30.83	65/65			
*P* (nmol/L)	WQF (2021)	0.41	0.16	0.61	0.18	30/30	Fixed	0.0%	<0.0001
	DLL (2021)	0.39	0.15	0.6	0.21	47/47			
	TYY (2021)	0.39	0.11	0.64	0.18	50/50			
	LN (2020)	0.41	0.16	0.61	0.19	88/87			
	ZYY (2021)	0.38	0.14	0.59	0.17	30/30			
LH (IU/L)	WQF (2021)	5.76	1.71	5.42	1.63	30/30	Random	95%	0.2740
	DLL (2021)	5.75	1.68	5.37	1.57	47/47			
	TYY (2021)	4.57	1.01	5.53	1.35	50/50			
	LN (2020)	5.76	1.64	5.82	1.71	88/87			
	ZYY (2021)	5.74	1.62	5.81	1.69	30/30			
	LDP (2022)	3.25	1.21	6.15	1.44	65/65			
FSH (IU/L)	WQF (2021)	5.52	1.73	5.69	1.62	30/30	Random	90%	0.0778
	DLL (2021)	5.51	1.68	5.72	1.59	47/47			
	TYY (2021)	4.45	1.02	5.55	1.13	50/50			
	LN (2020)	5.52	1.75	5.56	1.85	88/87			
	ZYY (2021)	5.51	1.73	5.54	1.83	30/30			
	LDP (2022)	5.11	1.25	7.44	1.83	65/65			
CA125 (IU/ml)	WQF (2021)	42.22	11.43	62.43	11.69	30/30	Random	91%	<0.0001
	DLL (2021)	42.16	11.58	62.56	11.78	47/47			
	MD (2017)	51.31	23.46	62.23	19.58	40/40			
	WM (2019)	42.02	11.65	63.25	12.08	85/85			
	BXH (2020)	42.01	9.32	63.36	11.43	53/53			
	LN (2020)	25.42	3.98	35.67	5.22	88/87			
	ZWX (2021)	41.06	10.32	53.97	11.08	38/37			
	LDP (2022)	18.33	10.62	25.74	10.36	65/65			
CA199 (IU/ml)	WQF (2021)	26.22	4.63	35.54	5.67	30/30	Fixed	31%	<0.0001
	LN (2020)	26.13	4.62	35.83	5.69	88/87			
	ZWX (2021)	40.36	9.88	54.13	10.75	38/37			
MPP2 (µg/L)	WQF (2021)	129.65	12.76	171.58	19.87	30/30	Fixed	0.0%	<0.0001
	DLL (2021)	129.81	13.05	171.69	20.06	47/47			
	TYY (2021)	128.44	13.02	171.12	18.99	50/50			
	LN (2020)	129.98	12.97	172.24	19.74	88/87			
Gal-3 (ng/L)	WQF (2021)	5.68	2.31	6.89	2.24	30/30	Fixed	0.0%	<0.0001
	DLL (2021)	5.7	2.25	6.94	2.16	47/47			
	TYY (2021)	5.32	2.02	6.86	2.04	50/50			
	LN (2020)	5.69	2.31	6.93	3.14	88/87			
VEGF (pg/ml)	WQF (2021)	85.65	50.67	121.58	82.84	30/30	Fixed	0.0%	<0.0001
	DLL (2021)	85.77	50.73	122.36	83.42	47/47			
	LN (2020)	85.78	53.46	121.85	89.37	88/87			
MPP9 (ng/L)	DLL (2021)	565.47	220.75	690.26	293.42	47/47	Fixed	0.0%	<0.0001
	TYY (2021)	566.54	236.23	680.12	282.12	50/50			
	LN (2020)	568.38	237.53	687.34	298.47	88/87			
Diameter (cm)	MD (2017)	2.46	1.04	2.97	1.22	40/40	Random	98%	0.0003
	WM (2019)	1.02	0.56	2.87	0.61	85/85			
	BXH (2020)	1.01	0.47	2.73	0.62	53/53			
	LN (2020)	0.69	0.25	1.25	0.31	88/87			
	ZWX (2021)	0.72	0.19	1.24	0.28	38/37			
	ZYY (2021)	0.67	0.23	1.23	0.29	30/30			
VAS	ZJ (2021)	2.03	0.2	2.79	0.26	43/43	Random	98%	<0.0005
	WM (2019)	2.94	0.87	5.63	0.89	85/85			
	BXH (2020)	2.92	0.69	5.64	0.98	53/53			
	LN (2020)	1.58	0.42	2.18	0.81	88/87			
	ZWX (2021)	1.17	0.26	2.39	0.47	38/37			
	ZYY (2021)	1.56	0.41	2.16	0.79	30/30			
ORT (days)	ZYH (2015)	13.27	2.49	18.58	2.91	63/63	Random	97%	0.0976
	JX (2021)	13.38	3.60	14.69	3.15	88/89			
MRT (days)	ZYH (2015)	26.37	4.12	33.61	3.29	63/63	Random	97%	0.0585
	JX (2021)	26.48	4.23	28.72	3.40	88/89			
ADR (%)	DLL (2021)	3		5		47/47	Fixed	13%	0.0097
	ZYH (2015)	6		17		63/63			
	WM (2019)	19		23		85/85			
	LN (2020)	6		14		88/87			
	LDP (2022)	5		4		65/65			

VAS, visual analogue scale; ORT, oviating recovery time; MRT, menses recovery time; ADR, adverse reaction.

### Grade evaluation of evidence quality

According to GRADE guidelines (45), the quality of the evidence for two outcomes of the rates of treatment effect and the rate of adverse effects, was assessed. The outcome indicators could be classified into four levels: high quality, medium quality, low quality, and very low quality. Evidence quality was measured using the following criteria: risk of bias, consistency, indirectness, imprecision, and publication bias. The summary of findings and the quality of evidence for study outcomes are presented in Table 4.

**Table 4:** GRADE rating of the quality of outcome.

Outcomes	Anticipated absolute effects* (95% CI)		Relative effect (95% CI)	№ of participants (studies)	Certainty of the evidence (GRADE)
	**Risk with DNG or TCM alone**	**Risk with DNG in combination with TCM**			
Cure rate	234 per 1,000	**323 per 1,000** (259 to 402)	**RR 1.38** (1.11 to 1.72)	831 (7 RCTs)	⊕◯◯◯ Very low^a,b,c^
Remarkable effect rate	372 per 1,000	**498 per 1,000** (439 to 565)	**RR 1.34** (1.18 to 1.52)	1,249 (11 RCTs)	⊕⊕⊕◯ Moderate^a^
Effective rate	252 per 1,000	**237 per 1,000** (196 to 287)	**RR 0.94** (0.78 to 1.14)	1,249 (11 RCTs)	⊕⊕⊕◯ Moderate^a^
Invalid rate	223 per 1,000	**51 per 1,000** (36 to 74)	**RR 0.23** (0.16 to 0.33)	1,249 (11 RCTs)	⊕⊕⊕◯ Moderate^a^
Rate of Adverse effects	182 per 1,000	**113 per 1,000** (78 to 162)	**RR 0.62** (0.43 to 0.89)	695 (5 RCTs)	⊕◯◯◯ Very low^a,b,d^

CI, confidence interval; RR, risk ratio.

* The risk in the intervention group (and its 95% confidence interval) is based on the assumed risk in the comparison group and the relative effect of the intervention (and its 95% CI).

^a^ None of the included studies reported blind intervention for patients.

^b^ Total number of events is less than 300.

^c^ There is publication bias.

^d^ Number of studies too small to assess the publication bias.

## Discussion

In this study, the major focus of our meta-analysis was on the effectiveness and safety of TCM in combination with DNG in the treatment of endometriosis. The results showed that the combination was significantly better than the drugs alone, in cure rate, remarkable effect rate, invalid rate and adverse effects. The same conclusions were drawn from the results of the subgroup analyses depending on the course of treatment, the different TCM combined with DNG, and the different control groups. In addition, the combination showed significant advantages in other outcome indicators after treatment: E2, P, CA-125, CA-199, MMP-2, MMP-9, Gal-3, VEGF, cyst size, and VAS were significantly lower in patients on the combination than in those on the drugs alone.

Herbal medicine has been used for centuries as a treatment for endometriosis-related dysmenorrhea, pelvic inflammation, and cysts (46). Due to the complex pathogenesis of endometriosis and the limited therapeutic effects, TCM has been used to treat patients' primary lesions and control their symptoms (47). TCM aims for a healthy circulation and the removal of blood stasis. This could be an important strategy for preventing endometriosis angiogenesis (48). At the same time, herbal remedies for endometriosis are designed to relieve blood stagnation and nourish the kidney (49). A number of TCM formulas have been used to treat endometriosis-associated pelvic inflammation, and they have resulted in satisfying results (50). For example, Hua Yu Xiao Zheng decoction (49), Wenjing decoction (51), Guizhi Fuling Capsule (also called Guizhi Fuling Wan) (52), Saffron (53), and Chinese medicine using Curcuma phaeocaulis Valeton (48). Among them, Guizhi Fuling Capsule was the most frequently used of all TCM for the treatment of endometriosis (54), which is also consistent with our inclusion study (4/13), its mechanisms of action mainly included improvement of hemodynamics, acesodyne and anti-inflammation (55). Some studies have demonstrated the effectiveness of Guizhi Fuling Capsule alone in the treatment of endometriosis (56). Overall and subgroup analyses of our study suggests that combination therapy is more effective in cure, remarkable effect, invalid and adverse effects rate. Thus suggests that combination TCM with DNG is an option for the treatment of endometriosis. However, contrary to the findings of our study, some study suggested that the combination of Guizhi Fuling Capsule with western medicines may have a negative impact on the treatment of endometriosis (56). Therefore, more research is needed to prove exactly how effective TCM in combination with existing western medicine is in treating endometriosis.

Oestrogen plays a key role in endometriosis (57). Endometriosis is characterized by an excess of estrogen, which can contribute to endometriotic lesions expanding faster (58). E2, which is the most active form of estrogen, works mainly through estrogen receptors (59). It can promote growth of endometriosis lesions in a hormone-dependent manner, both through systemic and local production (60). In our study, combination therapy significantly reduced E2 levels in patients compared to drugs alone. Studies have shown that the expression of VEGF, MMP2 and MMP9 in ectopic endometrium is significantly higher than that in eutopic endometrium, which also suggests that over-expression of angiogenic factors and metalloproteinases may be characteristic of those endometrium with the potential to transform into endometrial lesions (61). It has also been shown that VEGF, MMP-2 and MMP-9 are significantly elevated in the serum of patients with endometriosis (62). In contrast, CA-125 and CA-199 are significantly increased in the serum of patients with endometriosis and together with some other serological indicators can be used as a reference for the early diagnosis and staging of endometriosis (63, 64). Some study found that Gal-3 was over-expressed in all forms of endometriosis (65), it played an important role in the development of endometriosis and might be a target for endometriosis treatment (66). In our study, these indicators were significantly lower in the combination group than in the single drug group after treatment, which also suggests that the former is significantly better than the latter. In addition, LH, FSH, ovulation recovery time, and menses recovery time were not significantly different between the two groups after treatment, suggesting that the combination treatment did not impair ovarian function. This further supports that combination therapy maybe a good option.

Although peritoneal superficial lesions and ovarian endometriomas represent the majority of endometriotic implants within the pelvis, deep infiltrating endometriosis and extrapelvic endometriosis are the most challenging conditions to treat. Sometimes medical therapy is sufficient to reduce symptoms and signs (67, 68), however, in a large number of patients a complete eradication, with a nerve-sparing and vascular sparing approach (69, 70) is needed to restore normal pelvic anatomy and function. Many studies have suggested that DNG is effective in the treatment of deeply infiltrated endometriosis. It can prevent recurrence of post-operative DIE, control post-operative related pelvic pain (14, 15), reduce endometriotic lesions (71), reduce difficulties with intercourse and enhance sexual function (13), treat symptoms caused by rectosigmoid endometriosis (72), and improve quality of life (73). However, there are no studies regarding the use of TCM treatment for DIE. All the trials included in this study also did not classify endometriosis in detail, so it is not possible to know how effective herbal medicine combined with DNG is in treating DIE. Therefore, future clinical studies of DNG combined with TCM for DIE should be conducted.

This study had several limitations. First of all, the sample size was small, and the populations selected in this study were all Chinese. Therefore, our findings need to be validated in larger samples, other countries, and other ethnic groups. Second, not all RCTs reported their methods, which made assessing their methodological quality and judging their bias probability difficult; and there was publication bias in the cure rate data in our analysis, the quality of the evidence ranges from moderate to low. The reliability of the analysis results is therefore not high. Third, the lack of uniformity in herbal prescriptions may lead to potential bias. Fourth, all the literature included in the study was in Chinese owing to the geographical limitations of the use of Chinese medicine. Fifth, as the literature data does not provide more detailed data, it could not do further analysis according with endometriosis stage (refer to ASRM, ENZIAN) and localization (Superficial endometriosis, endometrioma, DIE).

In summary, the results of this meta-analysis suggest that TCM combined with DNG is superior to DNG or TCM alone and can be used as a complementary treatment after endometriosis treatment. TCM has potential for improving the clinical efficacy and reducing the side effects of DNG. A large, well-designed prospective study with a long-term approach is needed because of the limited number of included studies and the lack of precise methodology.

## Protocol assessment

Protocols can be accessed on PROSPERO (york.ac.uk).

## Data Availability

The original contributions presented in the study are included in the article/Supplementary Material, further inquiries can be directed to the corresponding author/s.
